# Evaluation of variant detection software for pooled next-generation sequence data

**DOI:** 10.1186/s12859-015-0624-y

**Published:** 2015-07-29

**Authors:** Howard W. Huang, NISC Comparative Sequencing Program, James C. Mullikin, Nancy F. Hansen

**Affiliations:** National Human Genome Research Institute, National Institutes of Health, Bethesda, MD USA

**Keywords:** Pooling, Sequencing, Algorithms

## Abstract

**Background:**

Despite the tremendous drop in the cost of nucleotide sequencing in recent years, many research projects still utilize sequencing of pools containing multiple samples for the detection of sequence variants as a cost saving measure. Various software tools exist to analyze these pooled sequence data, yet little has been reported on the relative accuracy and ease of use of these different programs.

**Results:**

In this manuscript we evaluate five different variant detection programs—The Genome Analysis Toolkit (GATK), CRISP, LoFreq, VarScan, and SNVer—with regard to their ability to detect variants in synthetically pooled Illumina sequencing data, by creating simulated pooled binary alignment/map (BAM) files using single-sample sequencing data from varying numbers of previously characterized samples at varying depths of coverage per sample. We report the overall runtimes and memory usage of each program, as well as each program’s sensitivity and specificity to detect known true variants.

**Conclusions:**

GATK, CRISP, and LoFreq all gave balanced accuracy of 80 % or greater for datasets with varying per-sample depth of coverage and numbers of samples per pool. VarScan and SNVer generally had balanced accuracy lower than 80 %. CRISP and LoFreq required up to four times less computational time and up to ten times less physical memory than GATK did, and without filtering, gave results with the highest sensitivity. VarScan and SNVer had generally lower false positive rates, but also significantly lower sensitivity than the other three programs.

**Electronic supplementary material:**

The online version of this article (doi:10.1186/s12859-015-0624-y) contains supplementary material, which is available to authorized users.

## Background

Due to recent advances in “next-generation” high-throughput sequencing (NGS) techniques, the cost of sequencing a human genome has fallen significantly over the past decade, from roughly 95 million dollars for the project that led to the human genome reference sequence to approximately five thousand dollars today [1]. Despite these large reductions in sequencing costs, it can still be prohibitively expensive to sequence and analyze a large number of samples individually. This makes it difficult to conduct the large scale sequencing studies necessary to detect and analyze rare variants, which have been suspected to contribute to a significant proportion of complex genetic diseases in humans [2].

Over the years, there has been discussion of the merits of pooling individual DNA samples together prior to sequencing [3, 4]. Pooling DNA, without including identifying index sequences, allows one to obtain and analyze genetic data from a larger number of individuals with only a fraction of the time and resources it would require to prepare and sequence each person individually. Increasing the number of human genomes and exomes analyzed through pooled sequencing could offer more comprehensive variant detection and better statistical power for variant association studies of genetic diseases. As a result, several programs have been written for the detection of variants in pooled sequencing data, including CRISP [5], SNVer [6], LoFreq [7], VarScan [8], and GATK’s Unified Genotyper [9].

However, there are a number of disadvantages in using pooled sequencing data for variant detection. First, any variant found using a one pool per sample scheme cannot be traced back to the original individual samples harboring that variant in the pooled sample. Furthermore, there is a risk of not detecting rare variants in pools with a large number of individuals. This is because each single variant would only be represented in approximately $\frac{1}{2n}$ of the pooled sample reads, where *n* is the number of diploid individuals in the pool. As a result, singletons, rare variants occurring only once in the pool, could have representation rates lower than the sequencing error rate if the pool has an especially large number of samples, and in the limit where the number of reads covering a site is less than the number of alleles, it becomes increasingly likely that a singleton variant will not be sequenced at all.

To resolve this issue, many variant detectors employ different combinations of various Bayesian and frequentist statistical models, read quality score analysis, and other known error patterns in Illumina and other NGS platforms’ sequencing reads to locate these singletons [5–9]. CRISP employs two methods to distinguish true variants from sequencing errors: to discover rare variants, it calculates a p-value against the null hypothesis of equal distribution of a proposed variant allele across all pools analyzed, and to identify common variants, it calculates a p-value for the null hypothesis of binomial distribution of sequencing error in each sample, requiring significance on both forward and reverse strand of the reference [5]. SNVer also employs binomial models of sequencing error rates and variant allele frequency to determine the p-value cutoff for true variants in a single pool, then uses the Simes method to create a “pooled p-value” from multiple pools [6]. To assign a p-value for each true variant, LoFreq models the distribution of variants in a sample as a Poisson-binomial distribution, then uses the phred-quality scores of each base call to model the sequencing error rate in its analysis [7]. VarScan selects and scans reads with the best alignment to a reference sequence to locate single nucleotide variants (SNVs) and indels [8]. Finally, GATK’s Unified Genotyper uses a Bayesian likelihood model to calculate the posterior probability of a variant at a particular position and determine allele frequencies in a pooled sample, given a user-specified number of alleles present per sample. Unlike the other programs, GATK provides the genotypes of each pool annotated with a phred-scaled confidence value [9].

In order for pooled genome sequencing to be ultimately feasible, a large proportion of variants and singletons must be retrievable from pooled read data. In addition, variant detectors must not report too large a proportion of false positives in order to provide results that are useful for subsequent studies. Therefore, it is valuable to perform an analysis of these variant detectors in order to better understand the potential benefits and tradeoffs of using pooled sequencing data. Determining the optimal variant detection programs and the best methods to run them could also prove useful for future genetic studies employing pooled sequencing techniques.

## Methods

### Generation of simulated pooled BAM files

To evaluate the five selected variant detection programs for accuracy, we ran each of them on pooled read data from two separately-generated datasets with known variants. First, we generated simulated pooled data using full-depth exome-captured Illumina HiSeq data from 256 individuals sequenced as part of the ClinSeq® Project [10]. In addition to aligning the read data with novoalign (http://www.novocraft.com) and removing PCR duplicate reads, we generated a “truth set” of variant call format, or VCF-formatted, files [11] specifying high confidence SNVs present in each individual, as well as browser extensible data, or BED, files containing the regions determined with high confidence to be nonvariant (homozygous reference), both using the bam2mpg variant caller [12]. To determine whether the alignment and preprocessing methods used prior to calling variants affects the accuracy of pooled variant detection, we also generated simulated pools from 64 lower depth exome-captured Illumina HiSeq reads from the 1000 Genomes Project [13], which had previously been aligned to the GRCh37 human reference with BWA, the Burroughs-Wheeler aligner [14] and processed with PCR duplicate removal, base quality recalibration, and realignment around known insertions and deletions according to currently accepted best practices [15].

To study the behavior of the programs we evaluated under different pooling scenarios, we created pools of varying depth of coverage and number of samples per pool, and then, when a program allowed it, analyzed these pools in groups of varying size. One program, LoFreq, only permitted the analysis of one pool of samples at a time, and another, CRISP, would only run on groups of pools. Pools were made by selecting random subsets of reads from the individual BAM files, reducing the number of reads from each individual from full coverage to 50 %, 25 %, or 12.5 % of the original total for that sample. These “titrated” BAM files were then merged into simulated pools of 4, 8, 16, or 32 samples using SamTools’s merge BAMs feature [16]. All possible non-overlapping groups of pools were then analyzed with each of the programs, allowing us to observe the variance of our accuracy measures across different sets of pools. Analyses were restricted to sequence data and variants from human chromosome 20 to decrease the time required to perform the analyses.

### Depth of coverage in pooled BAM files

The average number and standard deviation of total number of reads and average depth of coverage within targeted regions for each of the 256 individual ClinSeq BAM files and 64 individual 1000 Genomes BAM files are listed in Additional file 1: Table S1. The 256 ClinSeq samples had higher depth of coverage sequence data, in general, with an average of 70.2× read depth within regions targeted by the exome capture kit (standard deviation 21.1×), while the 64 1000 Genomes samples had an average depth of coverage of 42.0× (standard deviation 2.7×). Therefore, pools of ClinSeq samples that were sampled to contain 25 % of the original depth of coverage had an average of 70.2 times 0.25, or approximately 18× coverage per sample, whereas pools of 1000 Genomes samples sampled at 25 % of original coverage had only an average of 10.5× coverage per sample.

Although the relatively high variation in coverage per individual BAM file, especially for the ClinSeq samples, meant that the simulated BAM files had unequal read representation of each individual in each pool, this enabled us to test how well these programs can retrieve variants in the presence of this kind of variability. In fact, this distribution of read coverage among pooled samples simulates the real variability of actual pooled sequencing samples, since the sequence data for both the ClinSeq and the 1000 Genomes samples were generated by pooling indexed libraries prior to sequencing on the Illumina HiSeq platform. The sequence coverage obtained from pooling these identified libraries prior to sequencing can be expected to mimic the coverage for different libraries in a pool without identifying indexes.

### Installing and running variant detectors

We installed, ran, and evaluated results from the programs CRISP, SNVer, LoFreq, VarScan, and GATK’s Unified Genotyper. As these programs were written in different programming languages and have different software dependencies and options, we have included the details of each program’s installation and usage in the Additional file 2. Once we installed all of the programs, we ran them to see how much memory and processing time were required to analyze our pooled BAM files. SNVer, Varscan, and GATK had components written in Java, requiring us to request memory allocation prior to submitting jobs to the computer cluster, so we were more generous in providing memory to those programs. CRISP and LoFreq, which are written in C, required up to tenfold less memory than the other three, and therefore we were better able to determine the actual memory usage of these two programs.

We ran each of our selected programs on our two sets of pooled BAM files to locate single nucleotide variants (SNVs). GATK requires users to run a series of Picard tools in order to generate BAM indexes and process BAMs for its Unified Genotyper, and VarScan required users to pipe or input the BAM and reference files in pileup format, while no significant pre-processing was required by the other programs prior to running.

With the exception of LoFreq, all programs were also able to process BAM files from multiple different pools simultaneously. Following CRISP’s recommendations to run five or more pooled BAM files in each run [5], we ran every program except LoFreq with as many as eight pooled BAM files per analysis. When possible, we also ran each program on individual BAM files containing a single pool, to see how changing the number of BAM files per run affected SNV detection. For CRISP, which only allows processing of two or more pools, we compared results after running on more than two pools to results when running on two pools.

### Analyzing output data from variant detectors

All of the programs reported predicted variants in VCF format. Using the variants predicted by an independent method (bam2mpg) from the individually sequenced, full coverage samples, as well as the regions determined with high confidence to contain no variants in each sample, we checked each program’s pooled VCF file for accuracy and singleton detection rates. To do this, we first combined together all high-confidence SNVs in the single sample VCFs, and marked as singletons in each pool all SNVs that were present in only one sample from that pool. These variants constituted our “truth” set for a given pool. We also restricted our analysis for each pool or set of pools to the regions for which all samples in the pool had high confidence bam2mpg calls (either variant or homozygous reference), so that variants called in the pooled data which were unobserved in the individual samples could safely be assumed to be false positives (see Additional file 2 for more details). By comparing the pooled data calls to the variants present in the individual samples, we were able to calculate sensitivity as percentage of true variants detected, false positive rate as percentage of predicted variants which are false, and balanced accuracy as the mean of the sensitivity and one minus the false positive rate.

Although our analysis evaluated the accuracy of the five programs only with regard to single nucleotide variants, all five programs are also capable of predicting the locations of small insertion and deletion variants.

Since LoFreq could only process one pooled BAM file at a time, we merged single pool LoFreq VCFs and the corresponding true VCFs for each pool into groups of pools during the accuracy analysis of multi-pool runs. This way, the accuracy and singleton detection of LoFreq could be more fairly compared against the other programs’ runs on same-sized groups of pools. This particular analysis was also repeated for the other programs when they were processing individual pooled BAM files. Since CRISP had to process a minimum of two pooled BAM files per run, CRISP VCFs had to be grouped differently for results from two pools versus results from eight pools. To allow an even comparison between these two types of runs, four CRISP VCFs with two pools each were merged and compared against true variants for the relevant samples, and single CRISP VCFs with eight pools each were then compared against the same true variants. These groupings were structured so that data from both sets would contain the same numbers of individuals and variants per group during analysis.

To perform a ROC analysis for each program’s sensitivity to detect SNVs and singletons, we progressively filtered low quality scores or high p-values from each program’s output and measured sensitivity and number of false positives. This analysis was done on VCF output from the eight sample, 50 % coverage ClinSeq pools, with eight pools per program run, as well as the four sample, 50 % coverage Thousand Genomes pools, analyzed with four pools per program run. The score cutoffs were determined by calculating the range of quality scores produced by each program’s VCF, attempting to create a wide distribution of sensitivity and false positives calls for each program. In addition, we compared false positive and false discovery rates for each program to the rates implied by their reported quality scores.

### Ethics committee

Whole exome sequencing of samples from ClinSeq participants was approved by the National Human Genome Research Institute’s Institutional Review Board under protocol number 07-HG-0002.

## Results and discussion

### SNV detection results

Table 1 reports the sensitivity (%Sen), false positive rate (% FP), balanced accuracy (% Bal. Acc.), and singleton sensitivity rate (% Sing) for each program run on pools of different numbers of samples and coverage per sample. Scores reported in bold text represent better performance, while scores in non-bold text represent worse performance within each column.

**Table 1:** Program SNV Detection Results for (a) ClinSeq samples and (b) 1000 Genomes samples

a																
4 PooledSamples	%Sen	%FP	%BA	%SD	%Sen	%FP	%BA	%SD	%Sen	%FP	%BA	%SD	%Sen	%FP	%BA	%SD
	100 % Sample Covg				50 % Sample Covg				25 % Sample Covg				12.5 % Sample Covg			
CRISP	**99.2**	7.8	**95.7**	**98.9**	**97.3**	7.3	**95**	**94.2**	**88.9**	6.5	**91.2**	**76.5**	**71.3**	6.2	**82.5**	**44.9**
SNVer	81.9	**3.8**	89	72.4	74.9	**3**	85.9	59.1	62.7	**2.4**	80.1	37.3	48	**1.7**	73.2	16.6
LoFreq	**97.3**	8.3	**94.5**	**95.5**	**93**	**6.7**	**93.1**	**84.1**	**84**	**5.2**	**89.4**	**63.4**	**69**	**4.1**	**82.5**	**39.1**
VarScan	46.7	**0.1**	73.3	4.7	47.7	**0.1**	73.8	6.2	48.9	**0.1**	74.4	8.1	45	**0.3**	72.3	6.6
GATK	**99.7**	**6.9**	**96.4**	**99.4**	**98.7**	7.4	**95.7**	**96.7**	**94.7**	8	**93.3**	**86.3**	**85.7**	8.7	**88.5**	**65.1**
8 Pooled Samples	%Sen	%FP	%BA	%SD	%Sen	%FP	%BA	%SD	%Sen	%FP	%BA	%SD	%Sen	%FP	%BA	%SD
	100 % Sample Covg				50 % Sample Covg				25 % Sample Covg				12.5 % Sample Covg			
CRISP	**99.3**	7.8	**95.8**	**98.9**	**97.2**	7.4	**94.9**	**94.1**	**88.9**	6.8	**91.1**	**77.5**	**71.2**	6.7	**82.2**	**46.1**
SNVer	79.9	**3.6**	88.1	65.7	69.4	**3.2**	83.1	47.1	55.5	**2.8**	76.3	25.3	42.5	**2.4**	70	9.9
LoFreq	**96.7**	**7.3**	**94.7**	**93.1**	**91.8**	**6.5**	**92.7**	**79**	**82.8**	**5.4**	**88.7**	**56**	**70.7**	**4.3**	**83.2**	**31.8**
VarScan	28.8	**0.1**	64.4	0	29.2	**0.1**	64.5	0.1	29.8	**0.1**	64.8	0.1	30.4	**0.2**	65.1	0.3
GATK	**98.5**	8.6	**94.9**	**96.4**	**98**	8.5	**94.7**	**95.1**	**94**	10.1	**91.9**	**86**	**83.9**	11.4	**86.3**	**64**
16 Pooled Samples	%Sen	%FP	%BA	%SD	%Sen	%FP	%BA	%SD	%Sen	%FP	%BA	%SD	%Sen	%FP	%BA	%SD
	100 % Sample Covg				50 % Sample Covg				25 % Sample Covg				12.5 % Sample Covg			
CRISP	**99.1**	7.7	**95.7**	**98.5**	**96.7**	7.6	**94.6**	**93.3**	**87.7**	7	**90.4**	**76.6**	**69.3**	7	**81.2**	**46.3**
SNVer	66.9	**3.5**	81.7	42.9	53.7	**3.4**	75.1	23.8	42.4	**3.2**	69.6	10.8	33.2	**3**	65.1	3.6
LoFreq	**94.9**	6.4	**94.2**	**87.6**	**88.5**	6	**91.3**	**69.8**	**78.5**	5.5	**86.5**	**44.7**	**67**	4.8	**81.1**	**22.5**
VarScan	18.1	**0.1**	59	0	18.2	**0.1**	59	0	18.4	**0.1**	59.1	0	18.7	**0.1**	59.3	0
GATK	NA	NA	NA	NA	NA	NA	NA	NA	NA	NA	NA	NA	NA	NA	NA	NA
32 Pooled Samples	%Sen	%FP	%BA	%SD	%Sen	%FP	%BA	%SD	%Sen	%FP	%BA	%SD	%Sen	%FP	%BA	%SD
	100 % Sample Covg				50 % Sample Covg				25 % Sample Covg				12.5 % Sample Covg			
CRISP	**98.7**	7.9	**95.4**	**97.5**	**95.8**	7.6	**94.1**	**91.8**	**86**	7.1	**89.5**	**74.4**	**67.5**	7.3	**80.1**	**46.2**
SNVer	41.8	**4.2**	68.8	11	34.6	**4.2**	65.2	5	29.3	**3.8**	62.7	2.3	24.5	**3.8**	60.4	0.6
LoFreq	**90.7**	5.4	**92.7**	**77.5**	**82.2**	5.4	**88.4**	**55.7**	**71.1**	5.3	**82.9**	**31.2**	**60.5**	5.2	**77.6**	**13.8**
VarScan	11.4	**0**	55.7	0	11.5	**0.2**	55.7	0	11.5	**0.2**	55.7	0	11.6	**0.2**	55.7	0
GATK	NA	NA	NA	NA	NA	NA	NA	NA	NA	NA	NA	NA	NA	NA	NA	NA
b																
4 Pooled Samples	%Sen	%FP	%BA	%SD	%Sen	%FP	%BA	%SD	%Sen	%FP	%BA	%SD	%Sen	%FP	%BA	%SD
	100 % Sample Covg				50 % Sample Covg				25 % Sample Covg				12.5 % Sample Covg			
CRISP	**99.2**	4.4	**97.4**	**98.5**	**90.9**	4	**93.5**	**80.3**	**74.2**	3.7	**85.3**	**48.7**	**55.5**	3.3	**76.1**	**22.4**
SNVer	86.5	1.3	92.6	74.8	70.9	0.9	85	47.3	50.3	0.4	74.9	18.5	33.2	0.6	66.3	4.4
LoFreq	**96.8**	**0.4**	**98.2**	**94.1**	**87.3**	**0.2**	**93.5**	**73.9**	**71**	**0.1**	**85.5**	**44.3**	**48.9**	**0**	**74.4**	**19.7**
VarScan	44.6	**0**	72.3	0.9	45.1	**0**	72.5	3.2	42.3	**0**	71.2	3.8	33.4	**0**	66.7	1.4
GATK	**99.9**	**0.3**	**99.8**	**99.8**	**97.5**	**0.2**	**98.6**	**93.8**	**89.3**	**0.2**	**94.6**	**74.9**	**73.9**	**0.3**	**86.8**	**44.7**
8 Pooled Samples	%Sen	%FP	%BA	%SD	%Sen	%FP	%BA	%SD	%Sen	%FP	%BA	%SD	%Sen	%FP	%BA	%SD
	100 % Sample Covg				50 % Sample Covg				25 % Sample Covg				12.5 % Sample Covg			
CRISP	**99.2**	4.3	**97.5**	**98.4**	**91.3**	4.1	**93.6**	**81.4**	**75.5**	3.7	**85.9**	**50.4**	**57.7**	3.2	**77.2**	**23.8**
SNVer	80.5	2.1	89.2	62	61.8	1.8	80	30.1	44.5	0.8	71.8	9.3	30.6	0.8	64.9	1.6
LoFreq	**95.4**	**0.4**	**97.5**	**89.7**	**84.4**	**0.2**	**92.1**	**64**	**68.5**	**0.2**	**84.1**	**33.3**	**52.5**	**0**	**76.3**	**13.1**
VarScan	25.5	**0**	62.7	0	26	**0**	63	0	26.9	**0**	63.4	0	25.7	**0**	62.8	0.1
GATK	**99.6**	**1.5**	**99.1**	**99.1**	**97**	**0.7**	**98.1**	**92.6**	**88.4**	**0.5**	**93.9**	**72.9**	**72.9**	**0.4**	**86.2**	**42.7**
16 Pooled Samples	%Sen	%FP	%BA	%SD	%Sen	%FP	%BA	%SD	%Sen	%FP	%BA	%SD	%Sen	%FP	%BA	%SD
	100 % Sample Covg				50 % Sample Covg				25 % Sample Covg				12.5 % Sample Covg			
CRISP	**99.1**	4.2	**97.4**	**98.1**	**91.2**	4	**93.6**	**81.5**	**74.2**	3.5	**85.3**	**48.3**	**57.3**	3.3	**77**	**24.5**
SNVer	61.3	4.4	78.4	27.8	47.6	3.3	72.1	9.8	36.1	1.6	67.3	1.7	27.3	0.9	63.2	0.3
LoFreq	**91.8**	**0.6**	**95.6**	**80.8**	**78.9**	**0.2**	**89.3**	**50.3**	**63.2**	**0.1**	**81.5**	**22.4**	**49.2**	**0.2**	**74.5**	**7.4**
VarScan	14.9	**0**	57.4	0	15.1	**0**	57.6	0	15.3	**0**	57.6	0	15.6	**0**	57.8	0
GATK	NA	NA	NA	NA	NA	NA	NA	NA	NA	NA	NA	NA	NA	NA	NA	NA

GATK was unable to process the 16 or 32 pooled sample pools (see runtime results). Pools were run in groups of 8 for the ClinSeq samples and groups of 4 for the 1000 Genomes samples, except for LoFreq runs, which ran on individual pools, before grouping the results in sets of 8 (ClinSeq) or 4 (1000 Genomes) to calculating sensitivity, false positive rate, balanced accuracy, and singleton detection rate. Numbers reported in bold face represent the better performance values for each column

**Fig. 1:**
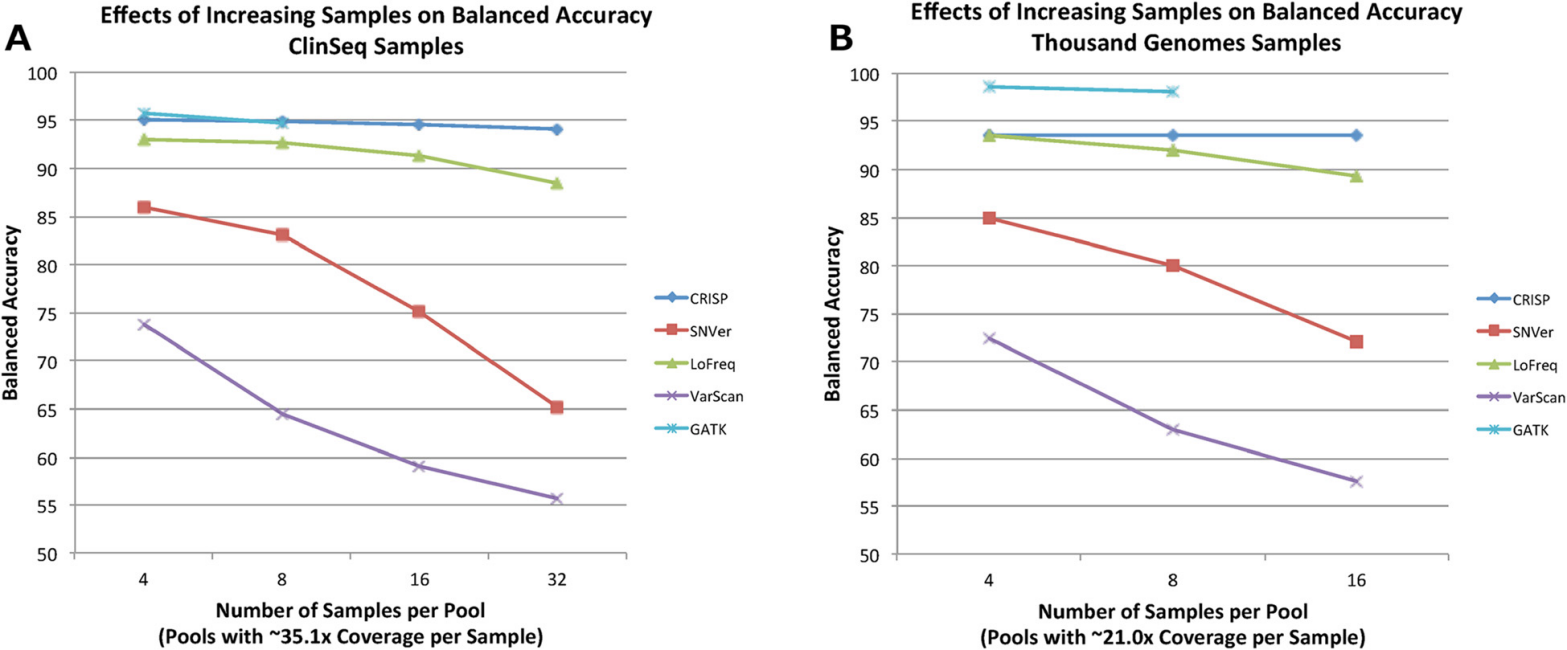
Effects of Pool Size on Program Balanced Accuracies. “Balanced accuracy” is defined as the mean of the sensitivity and 1 minus the false positive rate. No data point is reported for GATK with 16 or 32 samples because runs did not complete within a reasonable timeframe. Values are plotted for (**a**) ClinSeq and (**b**) Thousand Genomes pools containing read depth 50 % of a typical whole exome, which was 35.1x, on average, for ClinSeq samples and 21.0x, on average, for Thousand Genomes samples

**Fig. 2:**
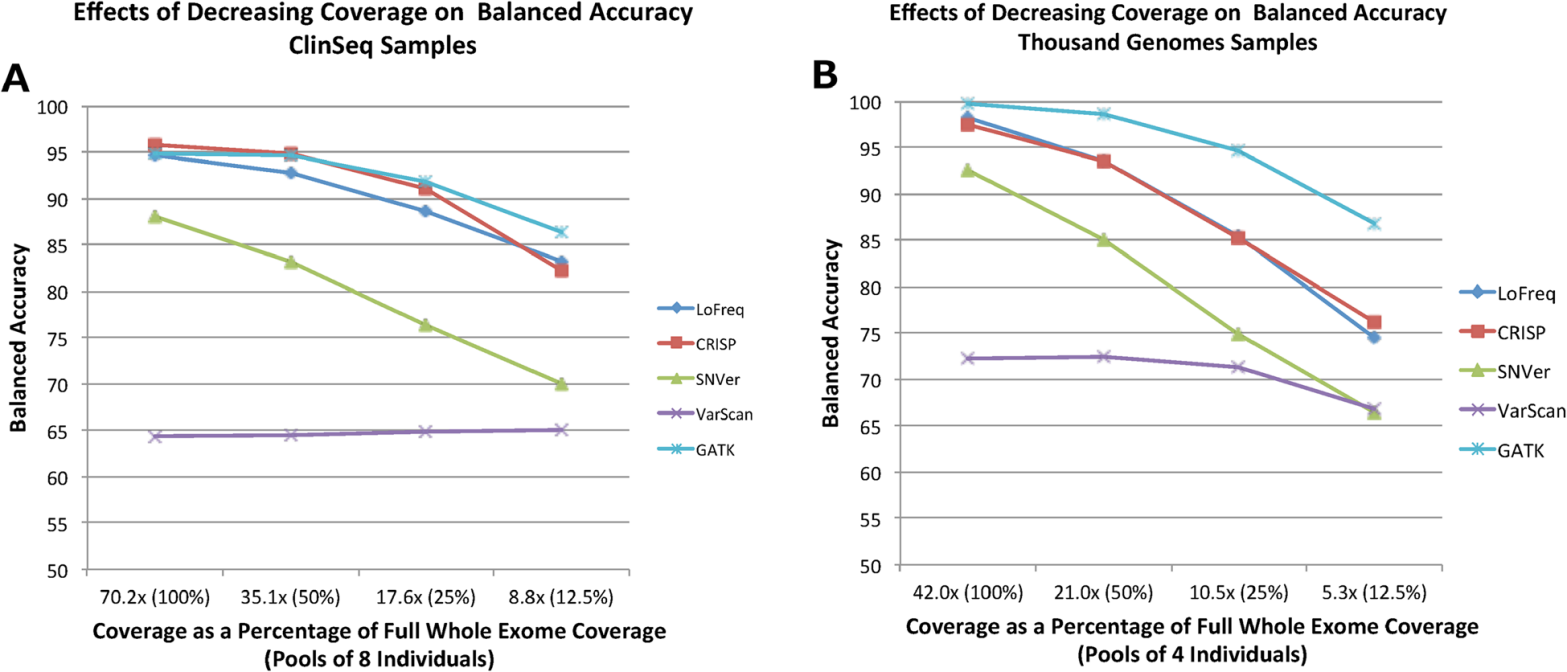
Effects of Pool Coverage on Program Balanced Accuracies. “Balanced accuracy” is defined as the mean of the sensitivity and 1 minus the false positive rate. Values are plotted for various fractions of “full coverage” for (**a**) ClinSeq pools containing eight individuals and (**b**) Thousand Genomes pools containing four individuals

In general, LoFreq, CRISP, and GATK achieved the highest balanced accuracy. While CRISP and GATK had higher sensitivities, LoFreq achieved good sensitivity with a lower percentage of false positive calls. GATK runs on BWA-aligned BAM files (from the Thousand Genomes dataset) resulted in lower false positive rates than GATK runs on novoalign-aligned BAM files (from the ClinSeq dataset). Figures 1 and 2 show the effects of increasing the number of individuals per pool and the coverage per sample, respectively, on balanced accuracy of each program. When the number of individuals per pool was increased, all programs except CRISP suffered from a higher rate of false positives, which decreased their overall balanced accuracy. Similarly, when the coverage for each individual was reduced, the sensitivity of each program suffered similar losses while their false positive calls improved.

While GATK had the best overall accuracy, ranging from 86 to nearly 100 %, when run on pools of four or eight samples, its run time increased significantly as the number of samples per pool increased. Since our GATK analyses of any number of pools with 16 samples each ran for greater than seven days without finishing, we decided not to report results for GATK in this scenario, for which CRISP had the best overall accuracy.

### Detection of rare variants

Table 1 also demonstrates the higher sensitivity of LoFreq, CRISP, and GATK for the detection of singleton variants in pools of four, eight, or sixteen samples. While this ability to detect rarer variants is a critical requirement in the analysis of pooled sequence data, GATK, especially, reported these variants along with a larger number of false positive calls, ranging up to nearly 11 % of all predicted variants when GATK is run on BAM files for the ClinSeq dataset. On the other hand, LoFreq attained high sensitivity for detecting singleton variants without a markedly increased false positive rate.

Since single samples displaying mosaicism, or somatic variants present in only a fraction of cells, also display variant alleles in small fractions of sequencing reads, the programs we evaluated could be run on sequence data to search for mosaic variants. Still, while pooled samples usually have a known number of chromosomes present, mosaic variants will be present in an unpredictable fraction of the DNA. All of the programs we evaluated, except for LoFreq, required the user to specify the number of chromosomes, or ploidy, present in the pool. Since the exact number of alleles present is unknown in a mosaic sample, LoFreq provides the convenience of not having to experiment by running programs with different values for this parameter, and may represent the best option for detection of mosaic variants.

### Filtering VCF output

**Fig. 3:**
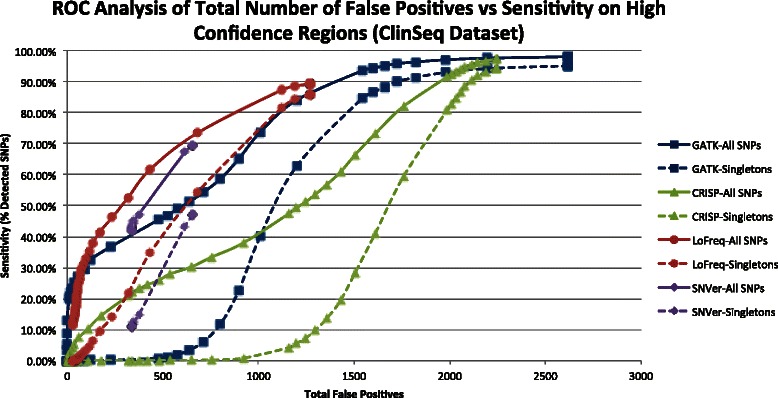
ROC Analysis on VCFs generated from ClinSeq eight sample, 50 % coverage pools with a total of 35.1x depth of coverage, on average, with eight pools per program run. For CRISP and GATK, quality score filtering was gradually increased on a logarithmic scale (0–100,100-1000,1000-10,000, etc.) to obtain a full range of sensitivity and false positive scores. LoFreq’s filtering was incremented logarithmically up to 1000, then by 100 s since its quality score range was smaller than those of the other programs. Many of SNVer’s P-values were extremely small (with reported p-values as low as 0), so maximum p-value filtering was set at values from 10^−10^ down to 10^−300^. Full details of score thresholds used are contained in the worksheet titled “Supp Table S2 Main Paper Figure S3” in the Additional file 1: Figure S3

Figure 3 shows the sensitivities and total false positive counts of each program’s eight sample 50 % coverage ClinSeq pool runs (eight pools per run, with average total coverage of 35.1x per pool) as variants were progressively filtered out using provided quality scores and p-values. Detailed values of quality thresholds and accuracy metrics for each program, as well as the corresponding graph for the Thousand Genomes dataset, are reported in Additional file 1: Table S2. As expected, singleton detection rates were more negatively impacted than overall program sensitivities during attempts to filter out false positives. For GATK, setting quality score cutoffs of roughly 70 to 90 led to moderate decreases in false positive calls without excessive losses in overall sensitivity and singleton detection rates. For CRISP and LoFreq, filtering was less beneficial and led to greater losses in sensitivity and rare variant detection rates than GATK. CRISP displayed a fairly linear relationship between overall sensitivity and false positive calls.

For SNVer, a large proportion of variants were reported with a p-value of 0. As a result, a large set of variants could not be filtered out. Similar to CRISP, SNVer has a fairly linear relationship between sensitivity and false positive calls and does not benefit significantly from filtering of variants with worse scores. VarScan, in general, had very low false positive calls, but also low sensitivity, which made filtration of its VCF files undesirable as well.

### Accuracy of quality scores

While Fig. 3 shows the range of sensitivity and false positive values each program attains, the actual quality scores, or equivalently, the predicted probability of a call being an error, used in filtering are not clear from the plot itself. Additional file 1: Table S2 gives the threshold scores used for the filtering done in Fig. 3, as well as the implied prediction error probabilities (for the phred-scaled quality scores reported by all programs but SNVer) or false discovery rates (for the p-values reported by SNVer). In general, reported quality scores for each of these programs are not predictive of the observed rate of false variant predictions. For example, LoFreq, GATK, and CRISP assign phred-scaled quality score values in the thousands, tens of thousands, and even hundreds of thousands, to variants, but clearly, the probability that a variant call with one of these scores is a false positive is higher than the near-zero error rates the quality values predict. For example, a phred-scaled quality score of 1000 corresponds to a probability that a variant call is false of 10^−100^, yet we observe in our analysis error rates ranging from 0 to 7 % in calls with a quality score of 1000 or higher. SNVer, which reports p-values rather than quality scores, also reports values that are not predictive of the actual false positive rate, or probability that an analyzed base without a variant will be predicted to have a variant. It assigns p-values as low as 10^−300^ to many calls, and these p-values are also far smaller than the observed error rates of these predicted high confidence variant calls. For example, in the Thousand Genomes set, five out of 3287 calls with p-values as small as 10^−300^ were found to be false.

### Comparing runs on individual pools versus groups of pools

Table 2 displays comparisons, for each program, of results obtained by submitting different numbers of pools to be analyzed together versus results obtained by running just one or two pools at a time. Surprisingly, submitting multiple pooled BAM files to each program did not result in significant improvements in accuracy as one might initially expect. Instead, at least one program (SNVer) displayed improved balanced accuracy when individual pooled BAM files were submitted. The fact that most programs showed little improvement in accuracy when analyzing large groups of pools simultaneously indicates that the added computational burden of processing a large dataset together may not be necessary to obtain good results.

**Table 2:** Effects of submitting multiple and individual pooled BAM files to each program

a				
Group Size	Sen%	FP%	BA%	SD%
CRISP-2 pools	97.8	10.5	93.7	95.3
CRISP-4 pools	96.1	7.5	94.3	91.6
CRISP-8 pools	97.2	7.4	94.9	94.1
SNVer-1 pool	72.4	3.3	84.6	52.9
SNVer-2 pools	71.4	3.3	84.1	51
SNVer-4 pools	70.4	3.3	83.6	49
SNVer-8 pools	69.4	3.2	83.1	47.1
VarScan-1 pool	29.3	0.1	64.6	0.1
VarScan-2 pools	29.3	0.1	64.6	0.1
VarScan-4 pools	29.3	0.1	64.6	0.1
VarScan-8 pools	29.2	0.1	64.5	0.1
GATK-1 pool	98.2	9.1	94.6	95.9
GATK-2 pools	98.2	9	94.6	95.8
GATK-4 pools	98.1	8.6	94.7	95.5
GATK-8 pools	98	8.5	94.7	95.1
b				
Group Size	Sen%	FP%	BA%	SD%
CRISP-2 pools	97.1	4.1	96.5	93.2
CRISP-4 pools	92.2	4	94.1	83.4
SNVer-1 pool	74.4	1	86.7	53.6
SNVer-2 pools	73.7	1	86.3	52.5
SNVer-4 pools	72.8	1	85.9	50.8
VarScan-1 pool	41	0	70.5	3.1
VarScan-2 pools	41	0	70.5	3.1
VarScan-4 pools	40.9	0	70.5	3.1
GATK-1 pool	98	0.2	98.9	95.2
GATK-2 pools	97.9	0.2	98.9	95.1
GATK-4 pools	97.9	0.2	98.9	95

In (a), all values were calculated using eight ClinSeq samples per pool with 35.1x average total coverage (50 % of typical full coverage for each sample). In (b), all values were calculated using four Thousand Genomes samples per pool with 21.0x average total coverage (50 % of typical full coverage for each sample)

### Analysis of false positives

To assess whether predictions the five programs made that were designated as false may in fact be false negatives in the truth sets we created with bam2mpg, we first determined to what degree the four of the programs’ false positive variants overlapped with each other. Because VarScan predicted so few false positives, we did not include it in this analysis. For pools of eight samples from the ClinSeq dataset at 12.5 % of normal coverage and analyzed in groups of eight (or individually, using LoFreq), we found that out of a total 2789 predicted false positives, only 70 were predicted by all four programs, and 2417 were predicted only by a single program (523 by CRISP, 1577 by GATK, 317 by LoFreq, and 0 by SNVer). A breakdown of the genomic locations of all 2789 false positives is given in Additional file 1: Table S3. In addition, an analysis of the variant allele frequencies of the 70 false positive predictions shared by all four programs revealed that 43 of these variants were found in at least one read in all 256 ClinSeq samples’ individual read datasets, which would be highly unlikely were they real variants, and 65 of them were not found in 50 % or more of reads in any of the 256 ClinSeq samples, which would also be highly unlikely were they true germline, diploid variants. Mean, standard deviation, minimum, and maximum values of the total depth of coverage and the variant allele frequency among the 256 samples for each of the 70 shared variants are given in Additional file 1: Table S4. Fifty-six of the 70 shared variants are located within one megabase of the chr20 centromeric sequence, indicating that they may actually be false positives resulting from mapping errors, since the centromeres consist mainly of repetitive sequence.

### Program memory allocation and runtimes

The approximate runtimes and memory allocated to each program are shown in Table 3. Overall, CRISP and LoFreq had the fastest runtimes and most efficient memory usage. Both programs were written in C. In contrast, GATK required roughly four times more time and up to ten times the amount of memory to run. Its runtime for analysis of eight sample pools was approximately 40 h, while its 16 samples pooled analyses were unable to finish running within a reasonable timeframe (greater than seven days).

**Table 3:** Program memory allocation and runtimes for pooled BAM files of 4, 8, and 16 ClinSeq samples, 35.1× average coverage each

Program	CPU Hours per BAM file	Memory Used/Provided
CRISP	<2 h	<150 Mb Used
SNVer	1 - 5 h	4 - 8 Gb Provided
LoFreq	1 - 5 h	~150 Mb Used
VarScan	2 - 5 h	6-8 GB Provided
GATK	8 h - +7 days	4 - 20 Gb Provided

Java programs required users to specify memory restrictions. Programs written in C were memory efficient and ran relatively quickly

## Conclusions

Based on simulated pooled data, LoFreq, CRISP, and GATK gave optimal balanced accuracy for most pooled datasets. Both CRISP and GATK were observed to have better sensitivity for singleton variants in pools than LoFreq when no filtering of calls is performed. However, LoFreq was found to have fewer false positives and was more flexible in terms of usage: it did not require users to specify sample ploidy, which makes the use of LoFreq more straightforward for analyzing data from mosaics. In addition, LoFreq has built-in features for detecting variants from somatic and cancer cell data, which are options worth pursuing given its high balanced accuracy for variant detection in large pools. In terms of runtime, memory usage, accuracy, and ease of usage, both LoFreq and CRISP were found to be better than GATK, and in fact, GATK was unable to process pools with 16 or more samples in a reasonable amount of time. Still, users wanting optimal sensitivity for smaller pools may find GATK to be worth the investment of increased time and memory requirements.

While this study did not evaluate the performance of these callers on true pooled samples, and only single nucleotide variant calls and not small insertion and deletion calls were assessed, the study can still serve as a useful starting point for users making choices about which software to run on pooled next-generation sequence data.

## Additional files

Additional file 1:
**Contains Supplementary Tables S1-S5, and data used to create figures. Huang BMC Bio Supplementary Data.**

Additional file 2:
**Contains details of methods not included in the main text.** Huang BMC Bio Supplementary Methods.

## Abbreviations

BAM: Binary alignment/map
BED: Browser extensible data
BWA: Burroughs-wheeler aligner
GATK: Genome analysis toolkit
NGS: Next generation sequencing
SNV: Single nucleotide variant
VCF: Variant call format
