# Supplementary material for: Evaluation of variant detection software for pooled next-generation sequence data
Source: BMC Bioinformatics. 2015 Jul 29;16:235. doi: 10.1186/s12859-015-0624-y (PMC4518579; doi:10.1186/s12859-015-0624-y)
Supplement: Additional file 2: — Contains details of methods not included in the main text. Huang BMC Bio Supplementary Methods. [file 12859_2015_624_MOESM2_ESM.docx]

**Supplementary Methods, Huang et al. “Evaluation of variant detection software for pooled next-generation sequence data”**

**Choice of programs to evaluate:**

In an attempt to make an unbiased choice of programs to evaluate, we first performed a literature search, choosing a set of eight tools that were either widely used and/or well presented in their publications. After making efforts to install and run all of these programs, we included in the study the five that we were able to install and run with what we considered to be a reasonable amount of effort. Programs we were not able to include in our analysis included vipr (Altmann et al., 2011), Syzygy (Rivas et al., 2011), and SNPSeeker (Druley et al., 2009).

**Installation and run parameters for evaluated programs:**

Genome Analysis Toolkit (GATK) version 2.8-1-g932cd3a and version 2.5 of the UCSC hg19 reference were downloaded from ftp.broadinstitute.org, and the UnifiedGenotyper was run using the “-l INFO” option. To perform pooled analysis, the “-ploidy” option was set to the number of chromosomes present in each pool. GATK “AddOrReplaceReadGroups” and Picard’s “SortSam” were run on BAM files prior to analysis.

Lofreq version 2.0.0-rc-1 (commit 2.0.0-rc-1-3-g63449f7) was installed and run in “call” mode.

CRISP version 0.7 was run setting the number of chromosomes in each pool with the “--poolsize” option.

SNVer version 0.5.3 was downloaded from http://snver.sourceforge.net/, and run setting the number of haploids and number of samples in a pool configuration file specified with the “-c” option. Default thresholds were used for variant detection.

VarScan version 2.3.6 was installed and “mpileup2snp” was run using default thresholds and settings for variant detection.

**Whole exome sequencing of 256 ClinSeq samples:**

Samples from 256 individuals from the ClinSeq study were sequenced at the NIH Intramural Sequencing Center in Rockville, Maryland. DNA samples were enriched for coding regions using Illumina’s “TruSeq” exome enrichment kit protocol and sequenced on HiSeq sequencers. Data were analyzed using Illumina’s CASAVA analysis package, and reads were then aligned to the UCSC hg19 reference sequence using novoalign version 2.08.02 (<http://novocraft.com>). PCR duplicate reads were removed using SamTools.

**Whole exome sequence datasets from 64 1000 genomes samples:**

BAM files for 64 HapMap samples listed in Supplementary Data Table S5 were downloaded from the 1000 Genomes ftp site at the addresses listed in <ftp://ftp.1000genomes.ebi.ac.uk/vol1/ftp/alignment_indices/20130502.exome.alignment.index> on January 29, 2015. To ensure uniformity of read length and capture type, only sequence reads of length 68 resulting from capture with Broad’s “Solexa-42316” kit were used.

**Determination of true variants from individual sample sequences**

In order to determine an individual sample’s high confidence variant genotypes, as well as high confidence genomic regions where no variant is present, we ran the program bam2mpg (Teer et al., 2010), which assigns a most probable genotype (MPG), as well as a log posterior probability score, to each base position at which at least one read is aligned. Positions with an MPG score greater than or equal to 10, as well as an MPG score greater than or equal to 0.5 times the depth of coverage at that position, have been validated against “Genome in a Bottle” genotypes (Zook et al., 2014) for the HapMap sample NA12878 in a separate study and found to have sensitivity of 99.99% (58463 out of 58467 true variants detected in high confidence regions) and false positive rate of 0.17% (100 out of 58563 predicted variants not in the Genome in a Bottle truth set). This false positive rate corresponds to roughly one single nucleotide variant false discovery every 696,000 bases.

Because we used these same methods to determine high confidence genotypes and locations of high confidence regions for the individual samples in our datasets, we can expect that there will be roughly 2 false positive single nucleotide variants and 1400 true positive single nucleotide variants in the 1.4 million average bases called on chr20 with high confidence in each 1000 genomes sample, and less than one false negative “missed” single nucleotide variant per 1000 genomes sample. For the ClinSeq samples, there are roughly 3 million average bases called on chr20 per sample, so we can expect closer to 4 false positive variants and 3000 true single nucleotide variants per sample, with still less than one false negative variant per sample. These accuracy rates make it highly unlikely that a variant predicted by the pooled sequence detection algorithms will be miscategorized as false when it is true, or true when it is false.

**References**

Altmann A, Weber P, Quast C, Rex-Haffner M, Binder EB, Muller-Myhsok, B: **vipR: variant identification in pooled DNA using R.** *Bioinformatics* 2011, **27**(13), i77-i84.

Druley TE, Vallania FLM, Wegner DJ, Varley KE, Knowles OL, Bonds JA, Robison SW, Doniger SW, Hamvas A, Cole FS, Fay JC, Mitra RD: **Quantification of rare allelic variants from pooled genomic DNA.** *Nat Methods* 2009, **6**(4), 263-265.

Rivas MA, Beaudoin M, Gardet A, Stevens C, Sharma Y, Zhang CK, Boucher G, Ripke S, Ellinghaus D, Burtt N, Fennell T, Kirby A, Latiano A, Goyette P, Green T, Halfvarson J, Haritunians T, Korn JM, Kuruvilla F, Lagace C, Neale B, Lo KS, Schumm P, Torkvist L, NIDDK IBDGC, United Kingdom Inflammatory Bowel Disease Genetics Consortium, International Inflammatory Bowel Disease Genetics Consortium, Dubinsky MC, Brant SR, Silverberg MS, Duerr RH, Altshuler D, Gabriel S, Lettre G, Franke A, D’Amato M, McGovern DP, Cho JH, Rioux JD, Xavier RJ, Daly MJ: **Deep resequencing of GWAS loci identifies independent rare variants associated with inflammatory bowel disease.** *Nat Genet* 2011, **43**(11), 1066-1073.

Teer JK, Bonnycastle LL, Chines PS, Hansen NF, Aoyama N, Swift AJ, Abaan HO, Albert TJ, NISC Comparative Sequencing Program, Margulies EH, Green ED, Collins FS, Mullikin JC, Biesecker LG: **Systematic comparison of three genomic enrichment methods for massively parallel DNA sequencing.** *Genome Res* 2010, **20**(10), 1420-1431.

Zook JM, Chapman B, Wang J, Mittelman D, Hofmann O, Hide W, Salit M: **Integrating human sequence data sets provides a resource of benchmark SNP and indel genotype calls.** *Nat Biotechnol* 2014, **32**(3), 246-251.
